# PU.1 controls the expression of long noncoding RNA HOTAIRM1 during granulocytic differentiation

**DOI:** 10.1186/s13045-016-0274-1

**Published:** 2016-05-04

**Authors:** Shuyong Wei, Ming Zhao, Xiaoling Wang, Yizhen Li, Kankan Wang

**Affiliations:** State Key Laboratory of Medical Genomics and Shanghai Institute of Hematology, Ruijin Hospital, Shanghai Jiao Tong University School of Medicine, 197 Ruijin Er Rd, Shanghai, 200025 China; Sino-French Research Center for Life Sciences and Genomics, Ruijin Hospital, Shanghai Jiao Tong University School of Medicine, Shanghai, 200025 China

**Keywords:** HOTAIRM1, PU.1, APL, PML-RARα, Transcriptional regulation

## Abstract

**Background:**

Long noncoding RNA HOX antisense intergenic RNA myeloid 1 (HOTAIRM1) has been characterized as a critical factor in all*-trans* retinoic acid (ATRA)-induced differentiation of acute promyelocytic leukemia (APL) cells. However, the essential transcription factor for gene expression of HOTAIRM1 is still unknown.

**Findings:**

Chromatin immunoprecipitation (ChIP) assays revealed that PU.1 constitutively bound to the regulatory region of *HOTAIRM1*. Co-expression of PU.1 led to the transactivation of the regulatory region of *HOTAIRM1* in a reporter assay. Detailed analysis showed that two PU.1 motifs, which were located around +1100 bp downstream of the transcriptional start site of the *HOTAIRM1* promoter, were responsible for the PU.1-dependent transactivation. The induction of HOTAIRM1 by ATRA was dependent on PU.1, and ectopic expression of PU.1 significantly up-regulated HOTAIRM1. Furthermore, low HOTAIRM1 expression was observed in APL cells, which was attributed to the reduced PU.1 expression rather than the repression by PML-RARα via the direct binding.

**Conclusion:**

PU.1 directly activates the expression of HOTAIRM1 through binding to the regulatory region of *HOTAIRM1* during granulocytic differentiation. The reduced PU.1 expression, rather than PML-RARα itself, results in the low expression of HOTAIRM1 in APL cells. Our findings enrich the knowledge on the regulation of lncRNAs and the underlying mechanisms of the abnormal expression of lncRNAs involved in APL.

**Electronic supplementary material:**

The online version of this article (doi:10.1186/s13045-016-0274-1) contains supplementary material, which is available to authorized users.

## Introduction

Recently, it has become increasingly clear that long noncoding RNAs (lncRNAs) function as a class of versatile regulators through interaction with DNA, RNA, and proteins to modulate gene expression [1–3]. Extensive studies have focused on the functions of lncRNAs in cells and demonstrated that lncRNAs modulate transcriptional regulation, regulate protein activities, play structural or organizational roles, and serve as precursors to small RNAs [4–7]. Thus, aberrant expression of lncRNAs is associated with disease development. However, the molecular mechanisms underlying the regulation of lncRNAs are still largely unknown.

HOX antisense intergenic RNA myeloid 1 (HOTAIRM1) is a myeloid-specific lncRNA, which is transcribed between the *HOXA1* and *HOXA2* genes. Functional studies have revealed that HOTAIRM1 is essential for myeloid differentiation. HOTAIRM1 knockdown impairs all*-trans* retinoic acid (ATRA)-driven granulocytic differentiation and attenuates the expression of differentiation-related genes including *ITGAM*, *CD18*, *HOXA1*, and *HOXA4* [8]. HOTAIRM1 knockdown can also result in a reduction of ATRA-induced cell cycle arrest at the transition from G1 to S phase [9]. A recent clinical study has investigated the clinical impact of HOTAIRM1 and demonstrated that high HOTAIRM1 expression is associated with poor prognosis in intermediate-risk acute myeloid leukemia (AML) patients [10]. In addition, the low expression of HOTAIRM1 is observed in acute promyelocytic leukemia (APL) [10]. Despite the importance of HOTAIRM1 in myeloid differentiation, the critical transcription factor regulating the expression of HOTAIRM1 has not been identified to date.

Transcription factor PU.1 is a master regulator in the control of myeloid differentiation [11–13]. PU.1 transcriptionally regulates the genes necessary for myeloid differentiation of granulocytes and monocytes, including granulocyte/macrophage colony-stimulating factor receptor (GM-CSFR) [14–16], CD11b [17], CD18 [18], and myeloperoxidase [19]. A tightly regulated level of PU.1 is essential for normal hematopoiesis. Dysregulation of PU.1 can lead to the development of AML [20–22]. Our previous work [23, 24] showed that, in the development of APL, both the targets and the expression level of PU.1 are inhibited by the oncogenic PML-RARα fusion protein, which is generated by the chromosomal translocation, t(15;17). Afterwards, a series of PU.1-regulated genes, such as genes encoding HCK [25] and immunoproteosomes [26], have been reported to contribute to APL leukemogenesis due to the dysregulation of their expression. During the treatment of APL, ATRA restores the expression of both PU.1 and its downstream target genes by degrading PML-RARα, thus relieving the differentiation block [27–29]. Moreover, genome-wide binding profiling of these APL-associated transcription factors, e.g., PML-RARα and PU.1, has demonstrated that the molecular consequences of these transcription factors are undoubtedly complex, as they associate with and likely regulate a relatively large number of diverse genes accounting for as much as 10–15 % of the genome [30]. Those target genes include not only protein-coding genes but also noncoding transcripts, especially lncRNAs.

In this study, using chromatin immunoprecipitation (ChIP) and luciferase reporter assays, we found that PU.1 bound to the regulatory region of *HOTAIRM1* in vivo and transactivated this region, which was mainly mediated through two PU.1 motifs around +1100 bp downstream to the transcription start site (TSS). Furthermore, PU.1 knockdown down-regulated HOTAIRM1 and PU.1 overexpression increased HOTAIRM1. Finally, we revealed that the reduced PU.1 expression was responsible for the low HOTAIRM1 expression in APL cells.

## Results

### The induction of HOTAIRM1 upon ATRA treatment requires intermediate protein synthesis

**Fig. 1:**
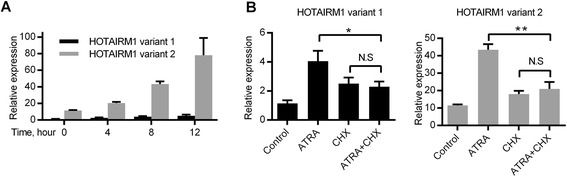
The induction of HOTAIRM1 upon ATRA treatment requires the intermediate protein synthesis. **a** The expression levels of two HOTAIRM1 variants at different time points following 1 μM ATRA treatment in NB4 cells. **b** The expression levels of two HOTAIRM1 variants in NB4 cells before and after ATRA treatment (1 μM) for 8 h, with or without 30 min CHX pretreatment (100 μg/mL). The relative expression level was calculated relative to that of variant 1 at 0 h. An average of two control genes (GAPDH and ACTB) was used for normalization. The data represent the mean of three replicates ± SD. *p < 0.05; **p < 0.01

It has been shown that HOTAIRM1 is up-regulated in APL cells upon exposure to ATRA and plays a critical role in myeloid differentiation [8]. Since HOTAIRM1 has two variants, we designed variant-specific primers to examine the expression of each variant in APL-derived NB4 cells upon ATRA treatment. As shown in Fig. 1a, HOTAIRM1 variant 2 was the major isoform that was expressed both before and after ATRA treatment. By comparison, HOTAIRM1 variant 1 was far less abundant than variant 2. Both variants were up-regulated upon ATRA treatment. To investigate whether the induction of HOTAIRM1 was a direct effect of ATRA, NB4 cells were pretreated with a translation inhibitor cycloheximide (CHX) for 30 min prior to the addition of ATRA to interfere with the translation process of newly synthesized proteins. As shown in Fig. 1b, CHX significantly impaired the up-regulation of both HOTAIRM1 variants by ATRA, suggesting that the induction of HOTAIRM1 requires newly synthesized intermediate protein(s).

### PU.1 directly binds to the regulatory region of *HOTAIRM1*

**Fig. 2:**
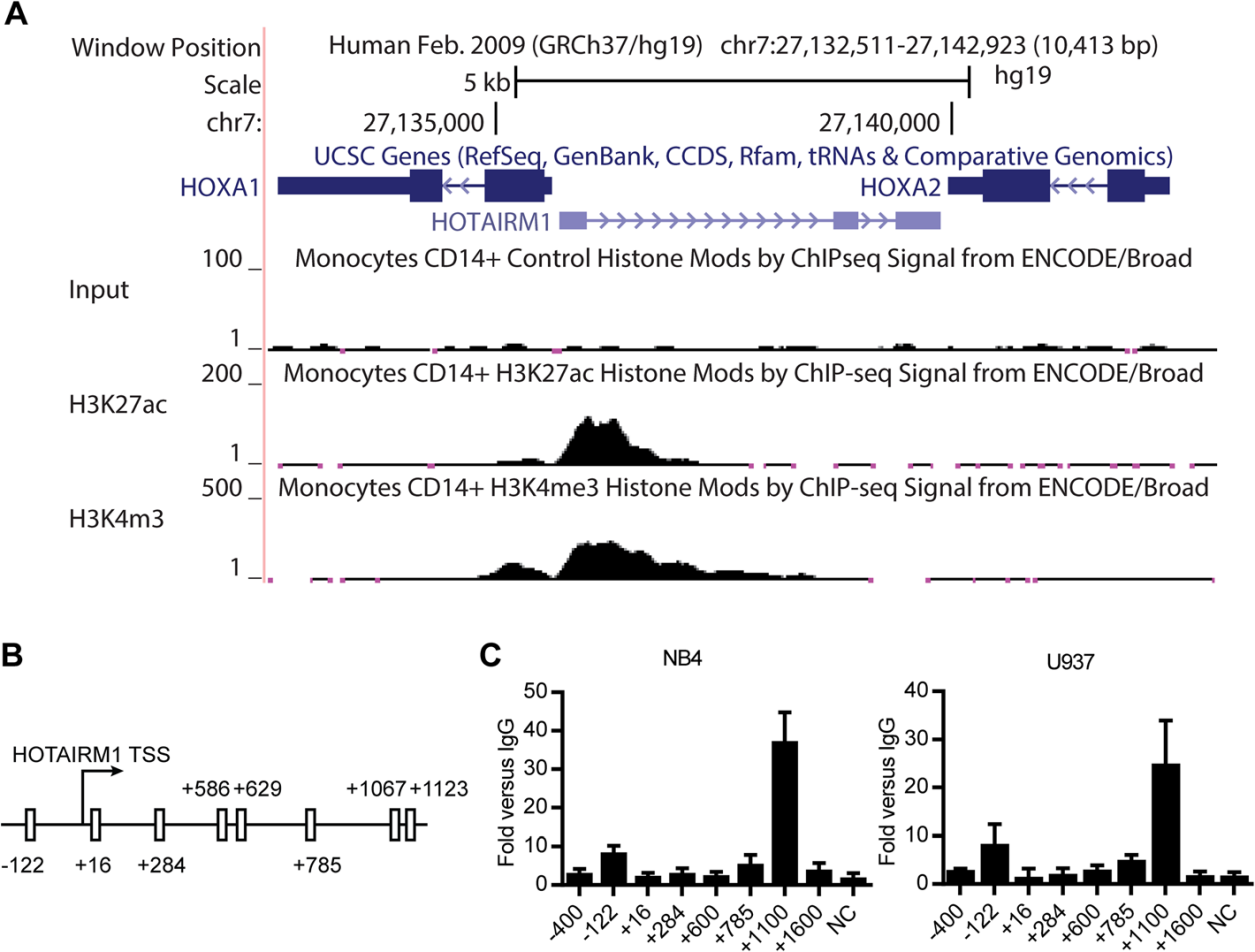
PU.1 directly binds to the regulatory region of *HOTAIRM1*. **a** Genome browser screen shot of the *HOTAIRM1* locus. Input (GSM1003475), H3K4me3 (GSM1003536), and H3K27Ac (GSM1003559) ChIP-seq signals of CD14+ monocytes were displayed in the UCSC genome browser. **b** Schematic representation of putative PU.1 binding sites around the promoter region of *HOTAIRM1*. **c** PU.1 ChIP-qPCR showing the enrichment of PU.1 in each putative binding site and the negative site in NB4 or U937 cells upon ATRA treatment for 24 h. The data represent the mean of three replicates ± SD

To search for the newly synthesized regulatory factors involved in the up-regulation of HOTAIRM1, we first identified the regulatory region responsible for the transcriptional regulation of *HOTAIRM1*, which can be defined using binding profiles of H3K4me3 and H3K27Ac. Due to the myeloid-specific expression of HOTAIRM1, we chose CD14+ monocytes (a myeloid lineage) for the analysis. We utilized the chromatin immunoprecipitation (ChIP)-seq data generated by the ENCODE Project Consortium [31]. The binding profiles were displayed using the UCSC Genome Browser (http://genome.ucsc.edu) [32]. As shown in Fig. 2a, the enrichment of H3K4me3 and H3K27Ac binding was found to be located from about −100 bp upstream of the transcription start site (TSS) to approximately +1200 bp downstream of the TSS. The data indicated that this region mediated the transcription of *HOTAIRM1* and this region was used in the following experiment and analysis. We then investigated the significantly enriched transcription factor binding sites within this regulatory region of *HOTAIRM1* using Matinspector [33]. Interestingly, we found eight putative binding sites for PU.1 (Fig. 2b), which is a hematopoietic transcription factor involved in myeloid differentiation and up-regulated upon ATRA treatment. To analyze the binding of PU.1, we performed ChIP assays in ATRA-treated NB4 cells. We designed six primer pairs to search for the in vivo binding of PU.1 around these eight PU.1-motif-containing sites. The closely located motifs were investigated using the same set of the primers. The primer pair “+600” covered motifs at +586 and +629 and the primer pair “+1100” covered motifs at +1067 and +1123. The regions upstream (−400) and downstream (+1600) of the regulatory region and an irrelevant region (NC) were used as negative controls. As shown in Fig. 2c, the position around +1100 bp downstream to the TSS showed dramatically significant enrichment of PU.1 binding in NB4 cells, and this was far more noticeable than the other putative sites. Additionally, we also detected the PU.1 binding in another myeloid cell line, U937, which belongs to a different AML subtype but can also undergo myeloid differentiation upon ATRA treatment. The similar PU.1 binding pattern was observed in ATRA-treated U937 cells, ruling out cell line-specific phenomena. The data together indicated that PU.1 directly bound to the regulatory region of *HOTAIRM1*.

### PU.1 transactivates *HOTAIRM1* through the +1100 region in the *HOTAIRM1* promoter

**Fig. 3:**
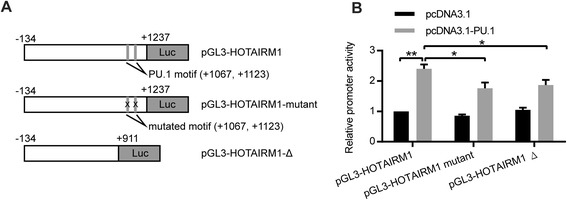
PU.1 transactivates *HOTAIRM1* through the +1100 region in the *HOTAIRM1* promoter. **a** Schematic representation of pGL3-HOTAIRM1 as well as its mutated and truncated forms. **b** PU.1-transactivated promoter activity of *HOTAIRM1*. Loss of major PU.1 binding sites significantly impaired PU.1 transactivation on the *HOTAIRM1* promoter. The luciferase promoters were co-transfected with equal amount of the pcDNA3.1 or pcDNA3.1-PU.1 vector to HEK-293T cells. The data represent the mean of three replicates ± SD. *p < 0.05; **p < 0.01

To further investigate whether PU.1 transactivates the regulatory region of *HOTAIRM1* through the PU.1 binding sites identified above, we employed the luciferase reporter assay in HEK-293T cells, which lack endogenous PU.1. We cloned a 1370 bp fragment of the regulatory region of *HOTAIRM1* spanning from −134 to +1237 into a luciferase reporter plasmid, which was designated as pGL3-HOTAIRM1 (Fig. 3a). We also included one mutant form of the complete regulatory region in which the core sequences of PU.1 sites at +1067 and +1123 were mutated from GGAA to CCAA, and one truncated construct spanning from −134 to +911 and lacking PU.1 sites on the +1100 region. As shown in Fig. 3b, co-expression of PU.1 resulted in significant activation of the full-length regulatory region of *HOTARIM1*. In contrast, the transactivation ability of PU.1 on the regulatory region of *HOTAIRM1* was significantly reduced in both PU.1-mutated and PU.1-truncated constructs (Fig. 3b). The results indicated that PU.1 could transactivate the *HOTAIRM1* promoter, and PU.1 motifs at +1067 and +1123 were essential for the transcriptional activation of *HOTAIRM1*.

### The induction of HOTAIRM1 is dependent on PU.1 during granulocytic differentiation

**Fig. 4:**
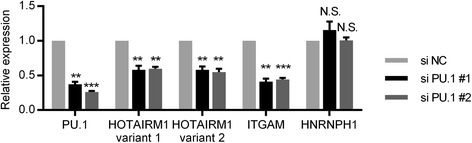
The induction of HOTAIRM1 is dependent on PU.1 during granulocytic differentiation. The expression of PU.1, HOTAIRM1, ITGAM, and HNRNPH1 after non-targeting or PU.1 siRNA transfection in NB4 cells, with ATRA treatment for 24 h. An average of two control genes (GAPDH and ACTB) was used for normalization. The data represent the mean of three replicates ± SD. *p < 0.05; **p < 0.01; ***p < 0.001

Next, we investigated the dependency of HOTAIRM1 on PU.1 in a hematopoietic context. To examine the in vivo regulation by PU.1, we silenced PU.1 in NB4 cells, followed by ATRA treatment for 24 h. To rule out off-target effects, two siRNAs against PU.1 were used and their knockdown efficiencies were around 63 and 74 %, respectively (Fig. 4). As a positive control, ITGAM (encoding myeloid integrin CD11b), whose expression is known to rely on PU.1 [17], showed a significant reduction. We used HNRNPH1 as negative control, as its expression level was not affected by the PU.1 level. Both HOTAIRM1 variants were significantly reduced in PU.1 knockdown cells compared with negative control cells (Fig. 4), suggesting that PU.1 was essential for the expression of HOTAIRM1 during granulocytic differentiation.

Additionally, we also detected the effect of arsenic trioxide (ATO), another effective agent used in APL therapy, on HOTAIRM1 expression. We found that ATO had almost no effect on the expression levels of HOTAIRM1 and PU.1 (Additional file 1: Figure S1), further supporting the above observation that the induction of HOTAIRM1 depended on the up-regulation of PU.1.

### Low HOTAIRM1 expression is observed in APL cells, which is attributed to the reduced PU.1 expression, rather than the direct binding and repression by PML-RARα

**Fig. 5:**
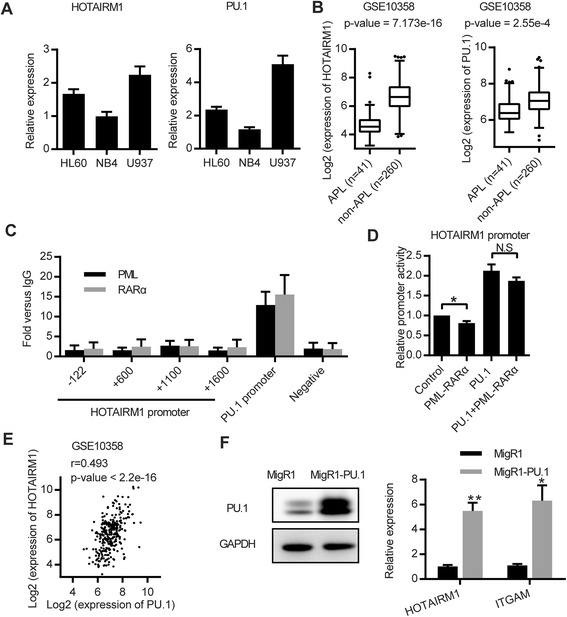
Low HOTAIRM1 expression is attributed to the reduced PU.1 expression. **a** HOTAIRM1 and PU.1 mRNA expression levels in representative AML cell lines. **b** Low levels of HOTAIRM1 and PU.1 in APL patients, as compared with non-APL patients (GSE10358). **c** ChIP-qPCR showing the binding of PML-RARα on the *HOTAIRM1* promoter, the PU.1 promoter (as a positive control), and the negative site. **d** The effect of PML-RARα on promoter activity of *HOTAIRM1*. The pGL3-HOTAIRM1 plasmid was co-transfected with 200 ng expression vectors (pcDNA3.1 alone, PML-RARα alone, PU.1 alone, PU.1 and PML-RARα together, respectively) into HEK-293T cells. **e** The Pearson correlation between PU.1 and HOTAIRM1 expression in an AML patient cohort (GSE10358). **f** The up-regulation of HOTAIRM1 after PU.1 overexpression. NB4 cells were harvested at 48 h post-transduction with the MigR1 empty vector or the MigR1-PU.1 vector. The expression level of PU.1 was detected by western blotting. The mRNA expression levels of HOTAIRM1 and ITGAM were detected by RT-qPCR. GAPDH was used for normalization. The data represent the mean of three replicates ± SD. *p < 0.05; **p < 0.01

Since HOTAIRM1 expression is up-regulated during ATRA-induced granulocytic differentiation of APL cells, we were interested in HOTAIRM1 expression in APL cells. We first compared the expression of HOTAIRM1 between APL cells and non-APL cells using AML cell lines. In APL patient-derived NB4 cells, the expression abundancy of both HOTAIRM1 and PU.1 was significantly lower than that in non-APL AML cells (U937 and HL60) (Fig. 5a). To validate this finding in a large cohort of patient samples, we utilized previously published transcriptome data [34] to compare their expression between APL patient samples and non-APL AML patient samples. As shown in Fig. 5b, compared to non-APL AML, HOTAIRM1 was noticeably lower in APL patients, which is consistent with a recent clinical study on AML patients [10]. Similarly, PU.1 was also lower in APL, supporting the above cell line-based results.

Low expression of HOTAIRM1 in APL could be due to several possibilities. First, since PML-RARα generally acts as a repressor on its direct target genes by recruiting co-repressors, such as HDAC1, N-coR, and SMRT [27], HOTAIRM1 expression might be repressed by the direct PML-RARα binding. To test this possibility, we first examined whether PML-RARα could directly bind to the regulatory region of *HOTAIRM1* using ChIP-qPCR assays. As shown in Fig. 5c, there was no enrichment of either PML or RARα binding around the regulatory region of *HOTAIRM1* in NB4 cells, indicating that PML-RARα did not directly bind to the *HOTAIRM1* promoter to repress its expression. The luciferase reporter assay also showed that PML-RARα had minimal impact on the promoter activity of *HOTAIRM1* (Fig. 5d). Both tests excluded the possibility of the direct binding and repression by PML-RARα.

Second, since we showed that *HOTAIRM1* was a PU.1 target gene and was activated by PU.1 (Figs. 2 and 3), and since PML-RARα is capable of repressing PU.1-regulated genes [23], another possibility might be that PML-RARα repressed PU.1-dependent transactivation of *HOTAIRM1*. This possibility was ruled out by the observation that PML-RARα led to a negligible change in PU.1-mediated transactivation through the luciferase reporter assay (Fig. 5d).

Third, low HOTAIRM1 expression could be attributed to the reduced PU.1 expression in APL since PU.1 expression is repressed by PML-RARα due to disruption of PU.1 autoregulation [23, 35]. To test this possibility, we first examined the correlation between PU.1 and HOTAIRM1 in a large cohort of AML patient samples [34]. We found a significant positive correlation between PU.1 expression and HOTAIRM1 expression (*r* = 0.493, *p* < 2.2e^−16^) (Fig. 5e). To support this finding, we rescued the PU.1 expression by over-expressing PU.1 in PML-RARα-positive NB4 cells. With PU.1 ectopic expression, HOTAIRM1 expression was increased more than fivefold in NB4 cells (Fig. 5f). This also provided an explanation for the up-regulation of HOTAIRM1 in NB4 cells upon ATRA treatment: ATRA restored the expression of PU.1 [29] and then the up-regulated PU.1 transactivated HOTAIRM1.

Taken together, the data suggested that low HOTAIRM1 expression in APL cells was attributed to the reduced PU.1 expression rather than repression by PML-RARα via the direct binding.

### Discussion

Recently, extensive studies have been carried out to understand the functions of lncRNAs, but fewer studies have focused on the regulation of lncRNAs. In this study, we reveal that the transcription factor PU.1 controls the expression of the lncRNA HOTAIRM1, which acts as a critical noncoding regulator in ATRA-induced granulocytic differentiation of APL cells. On the one hand, we showed that *HOTAIRM1* was not a direct ATRA-responsive gene, and the observed up-regulation of HOTAIRM1 induced by ATRA was a secondary event that was dependent on the expression of PU.1 activated by ATRA.

On the other hand, we observed low HOTAIRM1 expression in APL cells and revealed that low expression was attributed to the reduced PU.1 expression, rather than the direct binding and repression of PML-RARα. To support these observations, we provided the evidence that PU.1 constitutively bound to the regulatory region of *HOTAIRM1* to transactivate the promoter activity of *HOTAIRM1* and identified the two PU.1 motifs responsible for the PU.1-dependent transactivation. These findings enrich our knowledge on the regulation of lncRNAs and the underlying mechanisms of the abnormal expression of lncRNAs involved in APL.

Our first contribution is identifying *HOTAIRM1* as a novel target of PU.1. This finding implicates that there still exist unidentified PU.1 targets that are essential for myeloid differentiation. To date, most of the known PU.1 direct target genes participating in myeloid differentiation belong to protein-coding genes [36]. To the best of our knowledge, few have reported the role of PU.1 in the modulation of lncRNAs during myeloid differentiation. Our work presents an example of lncRNAs as the PU.1 target. In addition to *HOTAIRM1*, there are perhaps hundreds of lncRNA candidates potentially under the transcriptional control of PU.1, among which some are likely correlated with or necessary for myeloid differentiation. Systematic approaches like ChIP-seq and RNA-seq would be a powerful tool to globally identify these PU.1 candidate targets. In addition, we also identified the direct binding site of PU.1 at the +1100 downstream of the TSS of the *HOTAIRM1* promoter. It is worth mentioning that it was possible that the −122 region might also have a partial impact on the promoter activity of *HOTAIRM1*, although we focused on the +1067/+1123 locus in the luciferase reporter assay (Fig. 3b). The contribution from the −122 region was probably much less than the +1067/+1123 region, since the in vivo PU.1 binding signals at the +1067/+1123 locus were markedly higher than those at the −122 locus (Fig. 2c).

Second, our work suggests the role of HOTAIRM1 in the PU.1-mediated regulation network during myeloid differentiation. PU.1 regulates differentiation as well as cell cycle and proliferation. For example, exogenous PU.1 expression up-regulates endogenous PU.1 levels in progenitor cells, which is achieved by the induction of cell cycle lengthening, suggesting a positive feedback circuit between PU.1 and the cell cycle in the modulation of myeloid differentiation [37]. PU.1 occupies the *CDK6* gene promoter and directly stimulates *CDK6* transcription, coordinating proliferation and differentiation [38]. Our work found that *HOTAIRM1* was transcriptionally activated by PU.1 and their expression levels were significantly correlated in AML patients. Interestingly, HOTAIRM1 also regulates myeloid differentiation [8] and cell cycle arrest [9]. In addition, HOTAIRM1 modulates CD18 and ITGAM [8], which are known to be transcriptionally controlled by PU.1 [17, 18]. These findings suggest that HOTAIRM1 may participate in the downstream PU.1-dependent transcription regulation during myeloid differentiation. In another word, HOTAIRM1 may act as a downstream effector of PU.1 by regulating myeloid differentiation-associated genes and cell cycle-related genes.

Third, our research provides insights into the tissue specificity of HOTAIRM1. Tissue specificity is a characteristic of lncRNAs, which usually exhibit a more restricted expression pattern than protein-coding genes [39, 40]. Most recently, lncRNAs have been demonstrated to be dynamically expressed during erythropoiesis and these erythriod-specific transcripts are under the direct transcriptional control of core erythroid transcription factors GATA1, TAL1, or KLF1 [41]. In our work, we dissected the regulation control of *HOTAIRM1* by PU.1. Since PU.1 is prominent in myeloid cells, increases during myeloid differentiation, and achieves the highest level in mature granulocytes and monocytes [42], our results provide a reason to explain the high tissue specificity of HOTAIRM1 in myeloid cells. To fully explain the myeloid specificity of HOTAIRM1, more studies will be done to learn the whole picture of the combinational and coordinated regulation of HOTAIRM1 by other tissue-restricted transcription factors.

Finally, our work revealed the mechanism underlying the dysregulation of HOTAIRM1 in APL cells. PML-RARα is a critical factor required for the initiation and development of APL. At the transcriptional level, PML-RARα represses genes indispensable for normal granulocytic differentiation through multiple ways. First, PML-RARα interferes with the retinoid acid receptor α (RARα) signaling. As a potent transcriptional repressor, PML-RARα directly binds to the retinoic acid response elements (RAREs), recruiting co-repressor proteins such as DAXX, NcoR, and HDAC1 or methylating enzymes such as DNMT1 and DNMT3A, thereby leading to transcriptional repression [43]. Second, PML-RARα directly binds to PU.1-RAREh (RARE half) binding sites, physically interacts with PU.1, and thus inhibits PU.1-dependent transactivation of myeloid differentiation-associated genes, including *SPI1* (*PU.1*), *HCK*, *PSMB10*, and *IRF1* [23]. Our study provides an example of the third scenario, which receives less attention in the past compared to the former two scenarios. We did not observe the direct binding of PML-RARα in the regulatory region of *HOTAIRM1*. Instead, we found that the dysregulated expression level of HOTAIRM1 was due to the inhibition of PU.1, which suggested the PML-RARα-suppressive effect on *HOTAIRM1* was indirect. Also, this explains the noticeably low level of HOTAIRM1 in APL and suggests that the indirect suppression by PML-RARα can also be potent. Considering the indispensable role of HOTAIRM1 in myeloid differentiation [8], our work shows that PML-RARα-indirect inhibition of *HOTIARM1* contributes to the differentiation block in APL, which provides an additional mechanism for leukemogenesis in APL.

In conclusion, we identify the lncRNA *HOTAIRM1* as a novel PU.1-targeted gene and PU.1 mediates the myeloid-specific expression of HOTAIRM1. PU.1-dependent transactivation of *HOTAIRM1* relies on the two PU.1 motifs at the +1100 region of the *HOTAIRM1* promoter. The inhibition of PU.1 by PML-RARα contributes to the low HOTAIRM1 level in APL cells, which allows us to understand further how the oncofusion protein PML-RARα blocks myeloid cells at the promyelocytic stage during the development of APL.

## Materials and methods

### Cell culture and reagents

HEK-293T cells were cultured in DMEM (Gibco, Carlsbad, CA, USA) containing 10 % fetal bovine serum (Moregate Biotech, Bulimba, QLD, Australia), and NB4, HL60, and U937 cells were cultured in RPMI 1640 (Gibco) supplemented with 10 % fetal bovine serum. Cells were maintained at 37 °C in a 5 % CO_2_ incubator. All*-trans* retinoic acid (Sigma-Aldrich, St Louis, MO, USA) was added to NB4 or U937 cells at a final concentration of 1 μM.

### RNA extraction and qRT-PCR

Total RNA was extracted using an RNeasy Mini Kit (QIAGEN, Hilden, Germany) according to the manufacturer’s directions. Reverse transcription was performed using the SuperScript III Reverse Transcriptase (Invitrogen, Carlsbad, CA, USA). Real-time PCR was performed using the SYBR Green real-time PCR Master Mix (Toyobo, Osaka, Japan) in the Vii7 Real-Time PCR System (Applied Biosystems Inc., Foster City, CA, USA). All primers for quantitative reverse transcription-PCR (qRT-PCR) are listed in Additional file 1: Table S1.

### Plasmid construction

The regulatory region of *HOTAIRM1* was amplified using NB4 genomic DNA and then cloned to a pGL3-basic luciferase reporter vector (Promega, Madison, WI, USA). The PU.1-coding sequence was amplified using NB4 cDNA and then cloned to the MigR1 vector (Addgene).

### Chromatin immunoprecipitation assay

Chromatin immunoprecipitation (ChIP) was performed according to the Affymetrix protocol as previously described [44]. The following antibodies were used: anti-PU.1 (#2258; Cell Signaling Technology, Danvers, MA, USA), anti-RARα (C-20 X; Santa Cruz Biotechnology, Santa Cruz, CA, USA), and anti-PML (H238 X; Santa Cruz). All primers for ChIP-qPCR are listed in Additional file 1: Table S2.

### Luciferase reporter assay

Luciferase assays were conducted in HEK-293T cells as previously described [25]. Dual-luciferase reporter assays (Promega) were performed 24 h after transfection by Lipofectamine 2000 (Invitrogen). Renilla luciferase plasmids were used as an internal control for transfection efficiency correction.

### siRNA transfection

The siRNA oligos were transiently transfected to NB4 cells using Nucleofector Kit V (Amaxa Biosystems, Gaithersburg, MD, USA) according to the manufacturer’s instructions. PU.1-targeting siRNA oligos matching the sequences: CACUCAAGGCUCUUUGCUU (siPU.1 #1) and GAUGUUACAGGCGUGCAAA (siPU.1 #2), and a negative control siRNA (AGCGUGUAGCUAGCAGAGG), were used. After transfection for 24 h, cells were harvested for qRT-PCR.

### Retroviral transduction

The MigR1 vector (Addgene) was used to transduce NB4 cells for PU.1 overexpression. The preparation of retroviruses and transduction was done as previously described [45]. Briefly, the MigR1-PU.1 vector and the envelope plasmids were co-transfected into HEK-293T cells. After 48 h, retroviral supernatants were collected for transduction. NB4 cells were harvested at 48 h post-transduction.

### Gene expression analysis

The transcriptome data was provided by Tomasson et al. [34] (GSE10358). Robust multi-array average (RMA) [46] was used to calculate the expression level of HOTAIRM1 and PU.1.

## Additional file

Additional file 1:
**Figure S1.** Arsenic trioxide had minimal effect on the expression levels of HOTAIRM1 and PU.1. **Table S1:** Primers for RT-qPCR. **Table S2:** Primers for ChIP-qPCR. (DOCX 35 kb)

## Abbreviations

AML: acute myeloid leukemia
APL: acute promyelocytic leukemia
ATRA: all-*trans* retinoic acid
ChIP: chromatin immunoprecipitation
CHX: cycloheximide
lncRNA: long noncoding RNA
qRT-PCR: quantitative reverse transcription-PCR
TSS: transcription start site
