# Supplementary material for: PU.1 controls the expression of long noncoding RNA HOTAIRM1 during granulocytic differentiation
Source: J Hematol Oncol. 2016 May 4;9:44. doi: 10.1186/s13045-016-0274-1 (PMC4857283; doi:10.1186/s13045-016-0274-1)
Supplement: Additional file 1: — Figure S1. Arsenic trioxide had minimal effect on the expression levels of HOTAIRM1 and PU.1. Table S1: Primers for RT-qPCR. Table S2: Primers for ChIP-qPCR. (DOCX 35 kb) [file 13045_2016_274_MOESM1_ESM.docx]

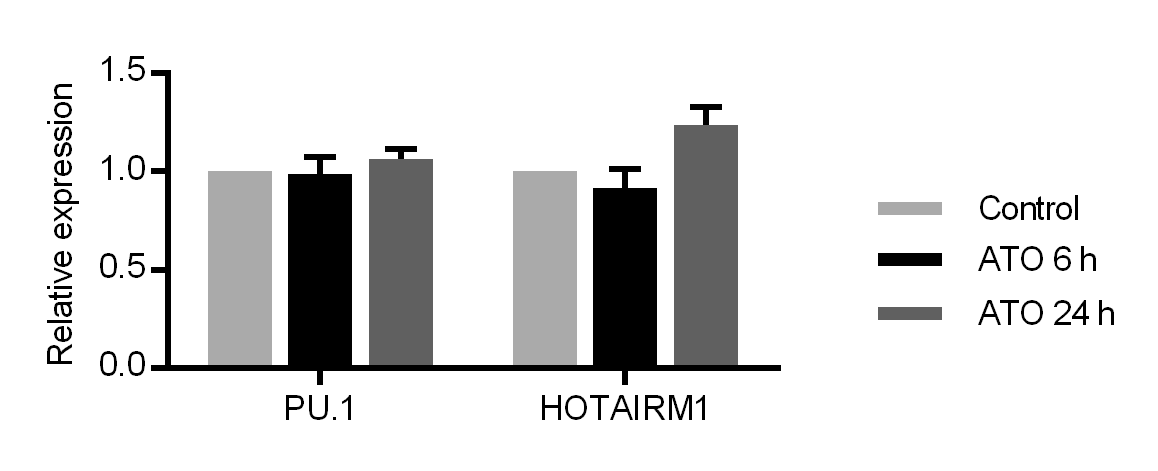


**Figure S1. Arsenic trioxide had minimal effect on the expression levels of HOTAIRM1 and PU.1.**

The expression levels of PU.1 and HOTAIRM1 were examined at 6 h and 24 h following 1 μM arsenic trioxide treatment in NB4 cells. GAPDH was used for normalization. The data represent the mean of three replicates ± SD.

**Table S1**

Primers for RT-qPCR:

| HOTAIRM1 variant 1 | Forward | ACATCGCGTTGTCATTGGAA |
| --- | --- | --- |
| HOTAIRM1 variant 1 | Reverse | TGGGTTCAGGCAAAACAGAC |
| HOTAIRM1 variant 2 | Forward | CTGGCGAGAGGTCTGTTTTG |
| HOTAIRM1 variant 2 | Reverse | AACACCCACATTTCAACCCC |
| HOTAIRM1 | Forward | CCCACCGTTCAATGAAAG |
| HOTAIRM1 | Reverse | GTTTCAAACACCCACATTTC |
| ITGAM | Forward | ACTGGTGAAGCCAATAACGCA |
| ITGAM | Reverse | TCCGTGATGACAACTAGGATCTT |
| HNRNPH1 | Forward | ATTCAAAATGGGGCTCAAGGT |
| HNRNPH1 | Reverse | GTGTCAGGACTATTTGGACCA |
| PU.1 | Forward | AAGTCCCAGTAATGGTCGCT |
| PU.1 | Reverse | AAGACCTGGTGCCCTATGAC |

**Table S2**

Primers for ChIP-qPCR:

| NC | Forward | TCTAAGGGGCAGCCTGATGT |
| --- | --- | --- |
| NC | Reverse | TCCTTCGCAAGGAAAAGAGC |
| PU.1 promoter | Forward | AAATCAGGAACTTGTGCTGGC |
| PU.1 promoter | Reverse | AGGAGTCCCGGTACTCACAGG |
| -400 | Forward | TCCAGAGTAAACAGCGGGAG |
| -400 | Reverse | ACTTCAGTGCGCCTTACAGC |
| -122 | Forward | CGTGACTGTGCCAACTTTCT |
| -122 | Reverse | CCTCTTGCGCACTGTACATT |
| +16 | Forward | CTTCCGCAGTGATGGATCA |
| +16 | Reverse | TGATCCATCACTGCGGAAG |
| +284 | Forward | CCCCACCGTTCAATGAAAGA |
| +284 | Reverse | CTCTGGCCCACTGATTCCC |
| +600 | Forward | CTCTTCAAGCTCGGAATGGA |
| +600 | Reverse | TTTCTTGCCCTCGTCTCCTT |
| +785 | Forward | CTTGGAGGGGCGGATGAG |
| +785 | Reverse | GAGCGACGAGAGAGGGAAG |
| +1100 | Forward | TAGTAGCTGAACTCCCTTCGT |
| +1100 | Reverse | ACTCTAGACGTGCAGCAGAA |
| +1600 | Forward | ACTCCAACCATGTGTACAATGT |
| +1600 | Reverse | CTAACGGGAGGAAAGGCTGA |
